# Lymph node fine needle Cytology in the staging and follow-up of Cutaneous Lymphomas

**DOI:** 10.1186/1471-2407-14-8

**Published:** 2014-01-06

**Authors:** Elena Vigliar, Immacolata Cozzolino, Marco Picardi, Anna Lucia Peluso, Laura Virginia Sosa Fernandez, Antonio Vetrani, Gerardo Botti, Fabrizio Pane, Carmine Selleri, Pio Zeppa

**Affiliations:** 1 Departments of Advanced Biomedical Sciences of Public Health, University of Naples "Federico II", Naples, Italy; 2 Advanced Biotechnologies, Biochemistry and Medical Biotechnologies, University of Naples “Federico II”, University of Naples, CEINGE, Naples, Italy; 3 Istituto Nazionale Tumori Fondazione "G. Pascale", Naples, Italy; 4 Department of Medicine and Surgery, Azienda Ospedaliera Universitaria “San Giovanni di Dio e Ruggi d’Aragona”, Largo città d’Ippocrate n.1, 84131 Salerno, (IT), Italy

**Keywords:** Lymph node, Cutaneous lymphoma, Fine needle cytology, Flow cytometry, PCR

## Abstract

**Background:**

Lymph nodal involvement is an important clinical-pathological sign in primary cutaneous lymphoma (PCL), as it marks the transformation/evolution of the disease from localized to systemic; therefore the surveillance of lymph nodes is important in the staging and follow up of PCL. Fine needle cytology (FNC) is widely used in the diagnosis of lymphadenopathies but has rarely been reported in PCL staging and follow-up. In this study an experience on reactive and neoplastic lymphadenopathies arisen in PCL and investigated by FNC, combined to ancillary techniques, is reported.

**Methods:**

Twenty-one lymph node FNC from as many PCL patients were retrieved; 17 patients had mycosis fungoides (MF) and 4 a primary cutaneous B-cell lymphoma (PBL). In all cases, rapid on site evaluation (ROSE) was performed and additional passes were used to perform flow cytometry (FC), immunocytochemistry (ICC) and/or polymerase chain reaction (PCR) to assess or rule out a possible clonality of the corresponding cell populations.

**Results:**

FNC combined with FC, ICC, and PCR identified 12 cases of reactive, non specific, hyperplasia (BRH), 4 dermatopathic lymphadenopathy (DL), 4 lymph nodal involvement by MF and 1 lymph nodal involvement by cutaneous B-cell lymphoma.

**Conclusions:**

FNC coupled with ancillary techniques is an effective tool to evaluate lymph node status in PCL patients, provided that ROSE and a rational usage of ancillary techniques is performed according to the clinical context and the available material. The method can be reasonably used as first line procedure in PCL staging and follow up, avoiding expensive and often ill tolerated biopsies when not strictly needed.

## Background

Primary cutaneous lymphomas (PCL) are the second most common extra-nodal non Hodgkin lymphomas (NHL) and represent a broad spectrum of distinct entities with different pathological presentations, clinical behaviours and treatment options [1]. Corresponding WHO/EORTC classification [1,2] accounts for approximately 20 distinct clinical-pathological entities, mainly divided into three diagnostic categories, namely cutaneous T-cell and/or NK-cell lymphoma, cutaneous B-cell lymphoma and precursor haematological neoplasm. As far as the staging of PCL is concerned, the TNM AJCC/UICC staging system [3,4] and the most recent TNM ISCL/EORTC staging system [5,6] identify three different parameters: the extension and characteristic of skin lesions, lymph nodal involvement and extra-cutaneous diffusion. Lymph nodal involvement represents an important clinical-pathological sign that marks the transformation/evolution of the disease from localized to systemic; therefore, PCL patients are closely observed for the possible development of palpable lymphadenopathies. Whereas the evidence of palpable lymph nodes alone determines the N1 stage in the histopathological staging of mycosis fungoides (MF) and Sézary syndrome (SS) [5], in clinical practice surgical excision and histological evaluation are generally applied to assess potential lymph nodal involvement by corresponding diseases [5]. Nonetheless, many PCL have a long standing clinical course [7] in which lymph nodal enlargement may arise at any time and for different reasons. For instance the incidence of dermatopathic lymphadenitis (DL) is higher in PCL patients than in others, but surgical excision is not easily performed or well accepted by the patients and surgical biopsy for diagnostic purposes alone might be considered an excessive solution/over-intervention in cases of unspecific benign reactive hyperplasia (BRH) or DL. Fine needle cytology (FNC), combined with different ancillary techniques, has gained a definitive role in the diagnosis of lymphadenopathies [8,9] although only few studies have explored a possible role for FNC in lymph nodal evaluation in the management of PCL [10,11]. These studies have investigated the possibilities of employing FNC exclusively in MF/SS [10,11] mainly focussing on PCR assessment of lymph nodes highly suspected or clinically involved by PCL. Nonetheless, we found that, in this specific clinical setting, lymph nodal FNC presents problems related to sampling, amount of cells obtained, variable cytological features, and the application of ancillary techniques. In our Institution FNC is generally requested for all enlarged lymph nodes that arise in patients suffering from any type of neoplasm, clinically or US suspected for malignancy. In this study we report our experience with FNC combined with ancillary techniques, on reactive and neoplastic lymphadenopathies arisen in PCL, including B-cell PCL. The aim of this study was to evaluate the possible role of lymph nodal FNC coupled with ancillary techniques in the staging and follow-up of PCL.

## Results

FNC combined with ancillary techniques (FC, ICC, PCR) provided the following diagnoses: 12 BRH, 4 DL, 4 lymph nodal involvement by MF and 1 by cutaneous B-cell lymphoma. Cytological features of the present series were quite variable and four main patterns were identified. The clinical, cytological, phonotypical and molecular data are summarized in Table 1.

**Table 1:** Clinical, cytological, phonotypical and molecular data of 21 lymph node fine needle cytology (FNC) of primary cutaneous lymphoma (PCL) patients

**Case n°**	**Clinical data**	**Lymph node site**	**FC**	**ICC**	**PCR**	**Cytological diagnosis**
1	MF/SS	Inguinal	Monoclonal	NP	NP	MF
2	MF	Axillary	Polyclonal	NP	NP	BRH
3	NHL B	Laterocervical	NC	NP	Polyclonal	BRH
4	NHL B	Axillary	NC	NP	NP	BRH
5	MF	Axillary	Polyclonal	NP	NP	DL
6	MF	Axillary	NC	UCHL1(CD45RO)+; CD3+	NP	MF
7	MF	Inguinal	Polyclonal	NP	NP	BRH
8	MF	Laterocervical	NC	NP	NP	BRH
9	MF	Inguinal	Polyclonal	NP	NP	BRH
10	NHL B	Axillary	NC	NP	NP	BRH
11	MF	Axillary	Polyclonal	NP	NP	DL
12	MF	Inguinal	NC	NP	NP	BRH
13	MF	Laterocervical	NC	CD3+; CD20+	NP	DL
14	MF	Laterocervical	Monoclonal	NP	Monoclonal	MF
15	NHL B	Axillary	Monoclonal	NP	NP	NHL B
16	MF	Axillary	Polyclonal	NP	NP	BRH
17	MF	Inguinal	polyclonal	NP	Polyclonal	DL
18	MF	Axillary	Polyclonal	NP	NP	BRH
19	MF	Inguinal	NC	UCHL1(CD45RO)+; CD3+	Monoclonal	MF
20	MF	Laterocervical	Polyclonal	NP	NP	BRH
21	MF	Laterocervical	Polyclonal	NP	NP	BRH

MF: mycosis fungoides; BRH: benign reactive non-specific hyperplasia; DL: dermatopathic lymphoadenopathy, NC: not contributive; NP: not performed.

### Reactive, non-specific hyperplasia

The FNC of reactive lymph nodes was quite similar in all cases, showing a variable mixture of normal cell type constituents and differing only in the amount of cells. The latter were mature small lymphocytes, follicular centre cells and reticular cells. Small lymphocytes were recognisable because of their size, round or elongated shape and dark compact chromatin. Follicular centre cells were medium sized and irregular in shape (centrocytes) or larger and roundish, with a bluish cytoplasm rime and nuclei with granular chromatin and two or more nucleoli (centroblasts and immunoblasts). Reticular cells were always present; nuclei were large with clumped chromatin and wide and pale cytoplasm. Vascular structures and phagocytic histiocytes were also present, conferring a polymorphous appearance to the smear. When a lymphadenopathy was determined by the expansion of the follicular centres, the smears showed numerous centrocytes and centroblasts intermingled with small mature lymphocytes, plasma cells and immunoblasts. In cases of interfollicular expansion the smears showed a prevalence of mature lymphocytes, plasma cells and immunoblasts; scattered epithelioid cells were occasionally present. In one case, groups of epithelioid cells organized in small granulomatous structures were detected and diagnosed.

### Dermatopathic lymphadenopathy

**Figure 1:**
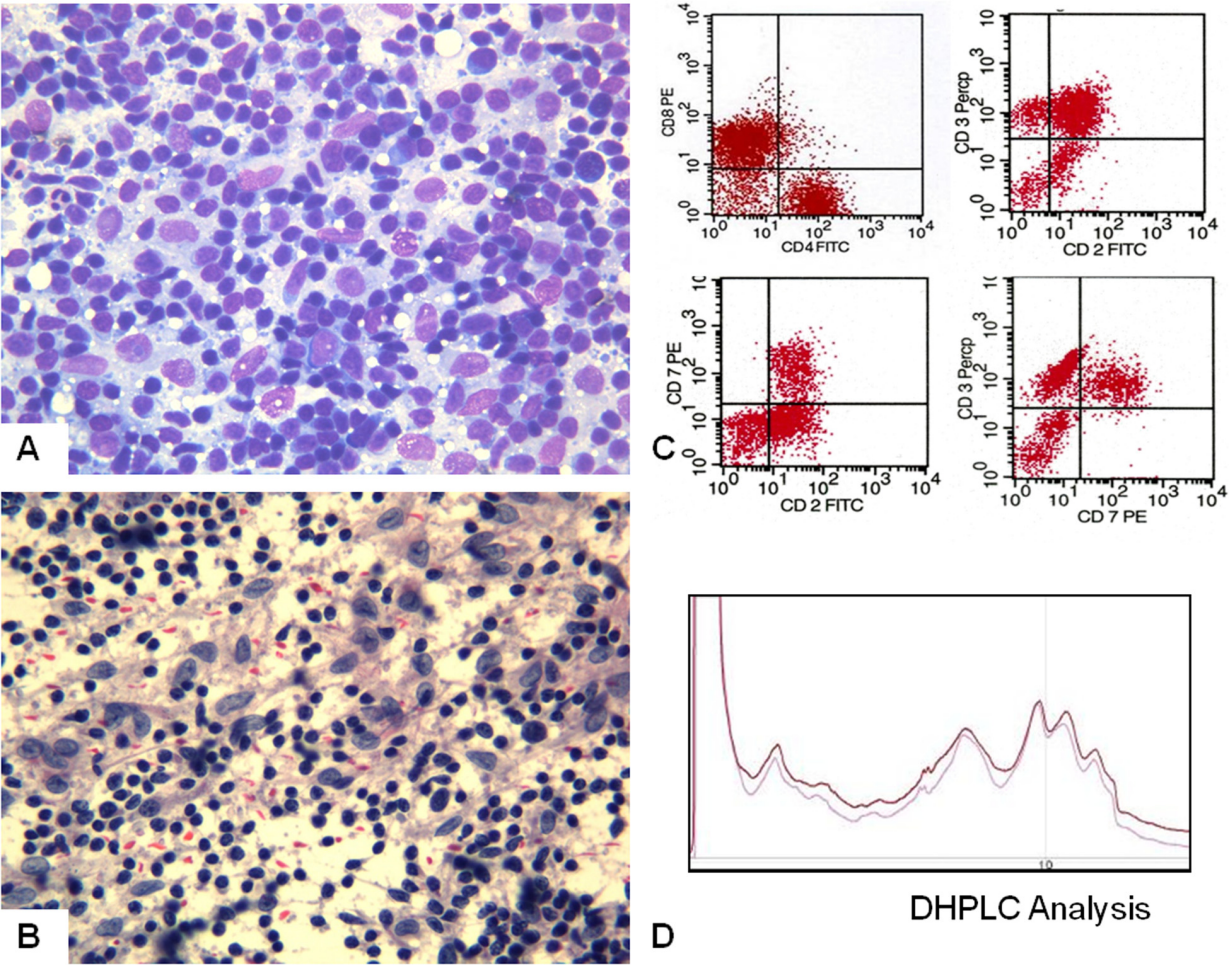
**Case 17, dermatopathic lymphadenopathy. A**: FNC smear of dermatopathic lymphadenopathy: the smear shows a polymorphous dispersed cell population represented by small lymphocytes, scattered histiocytes and occasional eosinophils (Diff Quik stain 430X). **B**: Small lymphocytes with compact chromatin and histiocytes with vesicular nuclei (Papanicolaou stain 430X). **C**: FC showing balanced CD4/CD8 ratio, and CD2/3/7 co-expression assessing the polyclonality of the T-cell population. **D**: DHPLC chromatogram of the TCRγ amplification product showing multiple peaks, in a shape similar to a Gaussian curve, assessing the polyclonal status.

Smears from lymph nodes with DL were quite cellular, with increased number of histiocytoid-dendritic cells (Figure 1A), sometimes clustered around vascular structures. These histiocytoid cells had abundant, pale blue cytoplasm, with indistinct cytoplasmic borders. The nuclei were elongated and vesicular with a fine chromatin pattern and irregular borders (Figure 1B). Nuclear grooves were rarely observed; macrophages containing brown melanin pigment were also observed. Mature lymphocytes, eosinophils and plasma cells were present in the background. Follicular centre cells were scantily represented in comparison with BRH cells.

### Mycosis fungoides

**Figure 2:**
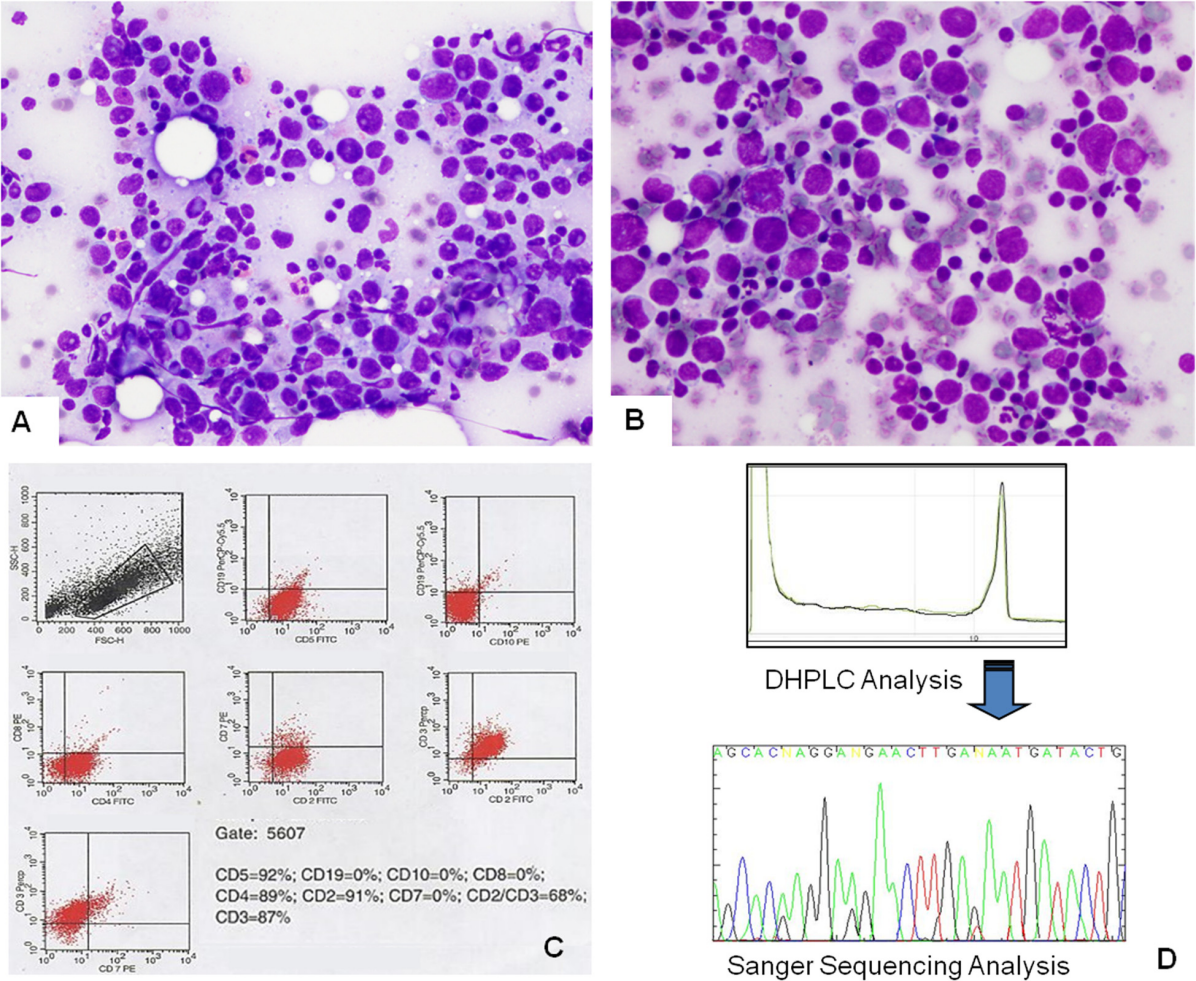
**Case14, lymph nodal involvement by MF. A**: FNC smear of lymph nodal involvement by MF showing an atypical cell population of medium sized cells with dense coarse chromatin. In the background there are mature lymphocytes and numerous eosinophils (Diff Quik stain 270X). **B**: Atypical lymphoid cells with nuclear irregularities, with lymphocytes and eosinophils in the background. **C**: FC showing the complete absence of CD8 cells and loss of CD7 in the same CD3/CD2 positive cells. **D**: DHPLC Chromatogram of the TCRγ amplification product showing a single peak assessing the monoclonal status. Sanger electropherogram of the TCRγ amplification product from the same case showing one type of sequence. Blast analysis indicated the homology between this sequence and the germline Vg4-Jg1/2 rearrangement as in the IMGT® databases.

FNC of the lymph nodes involved by MF showed a large number of medium sized cells with dense chromatin and irregular shape; the nucleoli were not easily detectable by either Papanicoplaou or Diff Quik stain (Figure 2A). Nuclear borders were irregular and seldom showed evident deep cleavages or histologically detectable foldings (Figure 2B). Background was polymorphous, consisting in small lymphocytes, reticular cells, and eosinophils (Figure 2A).

### B-cell lymphoma

**Figure 3:**
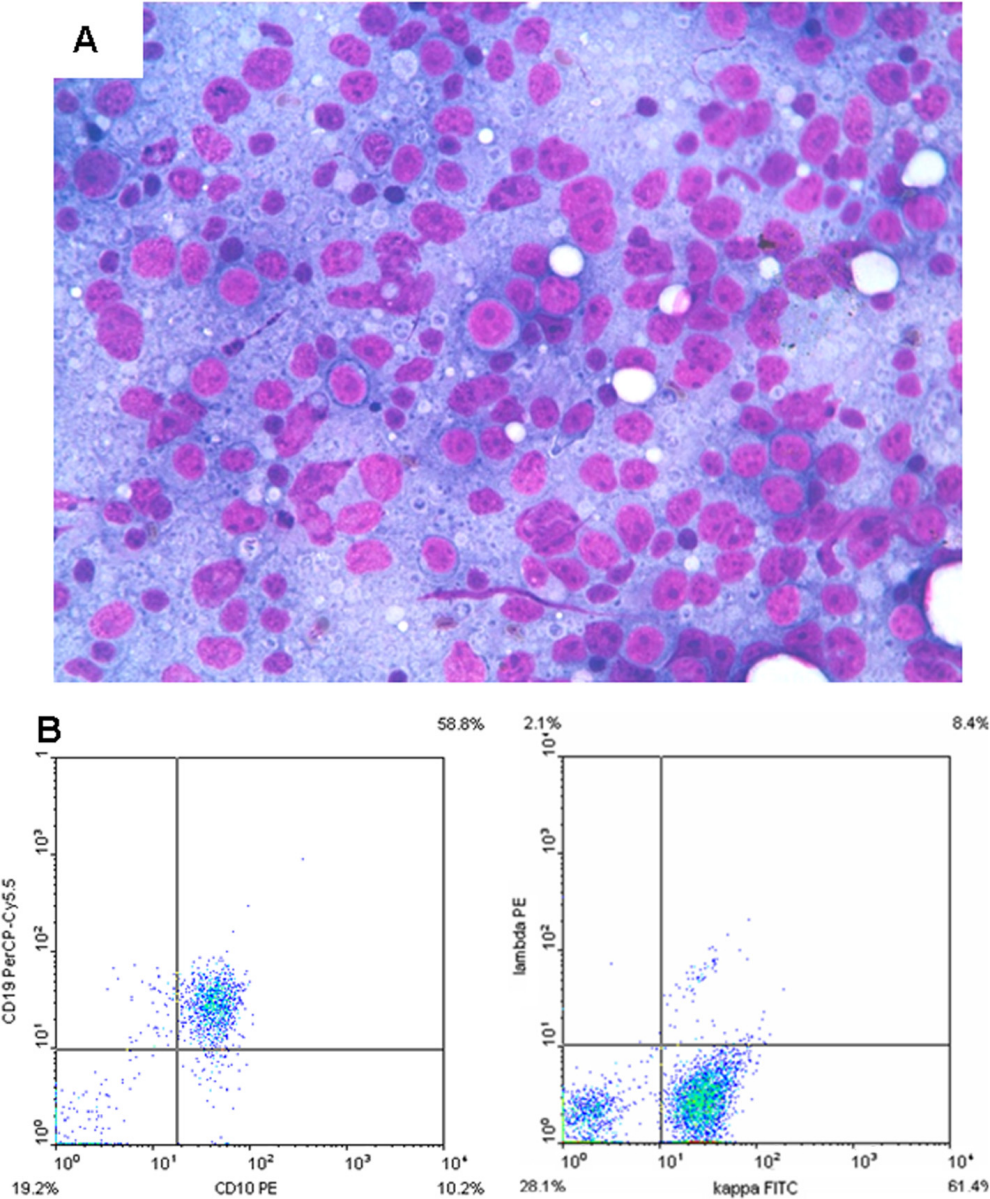
**Case 15, lymph nodal involvement from cutaneous B-cell lymphoma. A**: FNC smear of lymph nodal involvement from cutaneous B-cell lymphoma showing monomorphous large lymphoid cells with immature chromatin and two or more large nucleoli (Diff-Quik stain 430 X). **B**: FC showing CD10/19 co-expression and the kappa light chain restriction.

One case (case 15), diagnosed as B-cell lymphoma, had a history of large cell cutaneous B-cell lymphoma. The smear showed a relative monomorphous cell population of medium-large sized lymphoid cells with clumped chromatin and one or two nucleoli (Figure 3A); reticular cells, large follicular centre cells and macrophages were practically absent.

### Flow cytometry findings

FC assessment was successful in 13 cases, whereas, due to scanty cellularity, it was inadequate in the remaining 8. Of the adequate cases, 10 were reactive, 2 MF, and one B-cell NHL. All the cases of BRH and DL showed a normal CD4:CD8 ratio (3:1, 4:1) with T-cell surface antigens (CD2/CD3/CD7) co-expression (Figure 1C). B-cell antigens were also expressed, as was a variable amount of balanced light chains. In one of the cases (case 13) in which FC was not effective, ICC was performed showing proportional expression of CD3 and CD20. In particular, the large cells present in the smear were CD20 positive, confirming their B-cell origin. In two cases of lymph nodal involvement by MF (cases 1 and 14) FC showed an abnormal CD4/CD8 ratio (>10:1) and loss of CD7 expression (Figure 2C). In the remaining 2 cases (cases 6 and 19) FC was not effective, and ICC, performed on additional smears, showed UCHL1 and CD3 positivity in the medium-large sized cells with deep nuclear cleavages. In the lymph node involved by B-cell lymphoma (case 15), FC showed B-cell antigen over-expression, CD10 negativity and kappa light chain restriction (Figure 3B).

### TCRγ molecular analysis

In patients 3 and 17, the DHPLC analysis displayed a Gaussian distribution of amplified products (Figure 1D), while the blast analysis of the sequencing products obtained showed three productive rearrangements (Vγ4-Jγp, Vγ2-Jgp and Vγ4-Jg1) and one non rearranged Vγ8 segment, asserting the polyclonality of the corresponding T-cell population. Molecular analysis confirmed the monoclonal status in cases 14 and 19, showing single peaks at the DHPLC analysis (Figure 2D). Blast analysis of the sequencing products obtained showed the productive Vγ4-Jγ1/2 and the unproductive Vγ3-Jγ1/2 rearrangements, as illustrated in Figure 1B. Cytological diagnoses were confirmed by histology in positive cases, except in the B-cell lymphoma (case 15) – who presented clinical evidence of systemic disease, and in two benign DL cases (cases n. 5, 11) for whom ancillary techniques were not performed and there was disagreement with the clinical orientation. As for the remaining 14 negative cases, the patients underwent clinical and US follow-up. Follow-up time ranged between 5 years and 10 months, during which the cytological diagnoses were confirmed by reduction or persistence without increase in size or modifications of the US features of the corresponding lymph nodes.

## Discussion

Lymph node evaluation is an important step in the staging, prognosis and follow up of PCL, although there are differences in lymph nodal involvement between B-cell and T- cell lymphoma. Indeed, according to the TNM ISCL/EORTC staging system [5,6], in case of cutaneous B-cell lymphoma, microscopic evidence of regional lymph node involvement determines the transition from N0 to N1, N2 or N3 staging, depending on the peripheral region/s or central lymph node involvement respectively [6]. In case of MF and SS, the evaluation of the lymph nodes is different; in fact, because of the negative impact on survival rates of “palpable adenopathy”, their clinical evidence alone determines the transition from N0 to N1 staging [5]. This is a critical point in the management of MF/SS patients because the change in staging determines the need for systemic therapy; therefore, in clinical practice, histological evaluation can be requested. Criteria for lymph node removal in PCL staging are clinical and dimensional: the ISCL/EORTC revision [5] defines as clinically abnormal peripheral nodes in MF/SS those measuring 1.5 cm or more in the longest transverse diameter, or any palpable peripheral node, regardless of size, that on physical examination is firm, irregular, clustered, or fixed [5]. The 1.5-cm size is different from the 1-cm diameter node designated as abnormal in the ISCL/EORTC staging for non-MF/SS primary cutaneous lymphomas, since BRH or DL commonly occur in MF/SS, but arise less frequently in B-cell PCL [6]. These enlarged or clinically abnormal lymph nodes should also be evaluated by ultrasonography (US), computed tomography (CT) and 18 F-fluorodeoxyglucose positron emission tomography (FDG-PET) [5,6]. In cases of multiple lymph adenopathies, the guidelines suggest the biopsy of the cervical or axillary nodes first, and then the inguinal ones [5,12], according to the different probabilities of involvement. As for the lymph node status, both the Dutch and NCI-VA classifications [5] depended on their architecture and hence on histological evaluation. Therefore lymph node excision was the only considered procedure for this specific purpose. Nonetheless, simple histology too is not always effective; in the Fraser-Andrews’ study six of 19 patients with uninvolved lymph nodes or limited histological involvement (LN0-2) had a detectable T-cell clone at PCR investigation [13]. Moreover excisional lymph node biopsies are not always easily performed and may be complicated by sepsis in immunodepressed, especially erythrodermic patients [5]. Therefore the same guidelines suggest considering FNC as a possible diagnostic procedure for lymph node assessment possibly coupled with ancillary techniques [5]. Notwithstanding, relatively few studies have investigated the possible contribution of FNC in lymph nodal investigation in PCL [10,11] probably because of the problem of lymph nodal FNC false negatives that may arise in other neoplasm. In fact FNC false negatives mainly concerns lymph nodes involved by neoplasm that determine partial and sub-capsular metastases, such as breast carcinoma and melanoma [14,15]. In these cases, the needle may not succeed in sampling the specific involved areas of the lymph node, producing false negatives. Nonetheless, lymph node partial involvements is less frequently observed in haematological neoplasm [16] including cutaneous lymphoma. Moreover, according to the standardized cytological techniques of sampling, we moved the needle in different directions, during the FNC sampling, in order to reach different areas of the lymph node and to increase the probabilities of a representative sampling. Finally small cell clones may be not detected by FC but, in some CL, nuclear atypia are quite evident to be identified at the microscopic examination of the smears and by ICC, even though in small number. In the present study, although the basic approach of the technique was the same as that used in lymph nodal FNC from different contexts [8], specific problems were encountered mainly due to the different sites of development and clinical presentation. In fact, it was either impossible, or hardly possible, to perform FNC under US control on axillary lymph nodes due to their “mobility” and to the anatomical peculiarity of the axillary hollow which does not provide enough room for the US probe and the lateral needle holder, or even for the needle cap to guide the needle. Therefore, in our experience, palpable axillary lymph nodes were better approached by “pulling down” the node with the index and middle fingers and blocking it on the thoracic wall. One of the fingers was then used as a support for the needle while carrying out the sampling with or without aspiration. The second problem was presented by erythrodermic patients who are generally more sensitive than others, and often suffer from cold and have fragile skin that easily bleeds. Therefore lymph nodal sampling was performed quite rapidly on these patients, with no more than one additional pass and with careful capitalization of the material. For the above mentioned reasons, in our Department, ROSE is generally performed on lymph node FNC and always on PCL patients. This methodology, in addition to assessing the adequacy of the smear, allows a correct management of the material obtained according to the cytological features it identifies. In our study, for instance, for small-medium sized cell populations, cell suspension in buffered solution for FC and PCR was generally preferred. Conversely, in case of large cell populations additional alcohol fixed smears were preferred for ICC phenotypization. Therefore we believe that the “petals” largely compensate for the “thorns” that ROSE offers in this contexts [17]. In the study by Pai et al. [11] the cell block technique was used for ICC; in our laboratory cell block is highly effective in different cytopathological contexts but, in this specific series, since we did not obtain sufficient cellularity in the first two cases, we preferred to utilize residual material for other ancillary techniques. As for the positive cases, the cytological features observed were quite similar to those described by Pai et al. [11], and represent the only other extensive cytological description of lymph nodal MF/SS available, apart from the present one. However, we did not observe the predominant small-cell pattern as the one described by these authors. Ancillary techniques are basic tools in the cytological diagnosis of lymphoproliferative processes [8,9,18-20]; according to Galindo [10] and Pai [11] they are fundamental in this specific context, whereas we believe that some points should be discussed. In our experience, according to Galindo et al. [10] and Pai et al. [11], FC by CD4/CD8 ratio and the quantitative evaluation of CD7 still seems to be the most effective procedures, provided that a sufficient amount of cells is collected. Even in the case of lymph node involvement by B-cell PCL, FC was effective in demonstrating the B-cell phenotype and the corresponding light chain restriction as in the corresponding primary NHL. Nonetheless, in the present series FC was not effective in 8 out of 21 cases; this performance was definitely less effective than that observed in other FNC/FC lymph nodal series [8,9] and other procedures had to be applied. This finding hampers the comparison of the different applied techniques and should be not be surprising too; in fact in case of large diagnostic cell populations as the case of small cell B or T-NHL the procedure is highly effective [8,9]; conversely when diagnostic cells are relatively scanty and intermingled among benign reactive cells FC is proportionally less effective [8,9]. This is the case of Hodgkin lymphoma or anaplastic large cells and some high-grade T-cell lymphoma in which diagnostic cells are too scanty to be gated or being too large, they stick to the tubes of the equipment or get broken or lost determining false negative results. In these cases, conversely even few cells detected on light microscope may be sufficient for a definitive diagnosis, therefore other ancillary techniques had to be used. For instance, in 3 cases of scanty cellularity, ICC on additional smears was more effective than FC, allowing the identification of large atypical T-cell cells that were too scanty to be analyzed by FC. As for molecular testing, TCR-PCR is the most sensitive technique to assess T-cell clonality but also carries a relatively high rate of false positives [21]. In the study by Galindo et al. [10] there was total agreement between cytological FC and TCR on cytological samples in terms of both sensitivity and specificity. Conversely, in the same study, tissue/TCR was highly sensitive but less specific, as it detected three reactive lymph nodes as clonal [10]. Pai et al. [11] detected T-cell clonal population by TCR-γ PCR in two cases and, as expected, negativity in two Hodgkin lymphoma. In our cases TCR-PCR was performed on cytological material only in 4 cases and was also in agreement with the cytological/FC data. In addition to Galindo’s experience [10], a rate of false positive respectively of 3.6 and 5.4% for fresh and paraffin-embedded tissues was reported [21]. In a study performed on peripheral blood of MF and SS patients a 34% positivity rate on patients with benign cutaneous infiltrate was detected [22]. Therefore, as FNC is inevitably contaminated with peripheral blood, TCR-γ PCR should be carefully evaluated in this regard. Considering the dramatic evolution of molecular technologies, it is easy to foresee that highly sensitive and accurate procedures will shortly overcome specificity problems.

## Conclusion

Notwithstanding histology is the gold standard in lymph node evaluation and a larger comparative study between the two methods assessing their concordance is still compulsory, FNC might be considered as a first step procedure in PCL staging. FNC coupled with ROSE and ancillary techniques, utilized according to the clinical context and the available material, might be utilized to reinforce the negative diagnoses based on clinical and or imaging alone and possibly to avoid difficult biopsies in cases unequivocally positive.

## Methods

### Patients

From the files of the Cytopathology Service of the Department of Pathology, of the “Federico II” University of Naples, twenty-one lymph nodal FNC from PCL patients, performed over a 7-year period between January 2004 and December 2011, were retrieved. The study was approved by the Ethics Committee of the Istituto Nazionale Tumori Fondazione "G. Pascale" of Naples, Italy. At the time of FNC, patients were informed of the diagnostic procedure and its related risks; informed consent for the FNC performing, the diagnostic procedures and the scientific use of biological material was obtained from all the patients. No children were included in the study. Patients’ ages ranged from 39 to 71 years (mean age 55 yrs); 17 patients suffered from MF and 4 patients from a primary cutaneous B-cell lymphoma. The time from the first diagnosis to lymph nodal FNC ranged from three to 47 months. Sites of lymph nodes were: 6 cervical, 9 axillary, and 6 inguinal. Eight nodes were detected by US and 13 by clinical investigation; all were eventually US evaluated. The sizes ranged between 12 and 30 mm. Clinical data are summarized in Table 1.

At the time of FNC, patients were informed of the diagnostic procedure and its related risks; informed consent for the FNC performing, the diagnostic procedures and the scientific use of biological material was obtained from all the patients. No children were included in the study. Nine FNC were performed under US control and 12 by palpation and blocking the lymph node between the fingers. This was the case of 7 axillary lymph nodes in which US assisted FNC was difficult or impossible. Two patients showed different degrees of exfoliative erithroderma; therefore extreme care was taken in performing FNC in their case. Moreover in these patients the procedure was troubled by variable degree of pain on palpation, sensations of cold, and bleeding disproportionate to the gauge of the needle. According to the standardized cytological technique of sampling, we moved the needle in different directions during the FNA in order to reach different areas of the lymph node and to increase the probabilities of a representative sampling. The first smear was Diff-Quik stained for rapid on-site evaluation (ROSE) [17] of the adequacy of the sample; the remaining material left in the hub of the needle was carefully flushed with phosphate-buffered saline solution (PBS) and added, when necessary to a second pass in cases of scanty cellularity and used for flow cytometry (FC). When possible and according to ROSE cytological features, additional alcohol-fixed smears were used for Papanicolaou stain or conventional immunocytochemistry (ICC). In four cases a further pass was suspended in RNAlater TM for molecular investigation. In two cases residual cell suspensions were used to prepare cell-blocks but sections with sufficient cellularity were not obtained.

### Flow cytometry

Cell suspensions were processed within two hours; they were washed twice by centrifugation for 4’ at 2500 rpm, after which the supernatant was removed and added to 400 μL of PBS. When a sufficient amount of cells was available, the final suspension was divided into four or more tubes. One or two tubes of cell suspensions were stored until the end of the procedure in order to have additional material available in cases of unsatisfactory results or if further tests were needed. All the samples were then incubated for 15 minutes in the dark with 10 μL of the following basic combinations of phycoerythrin (PE), perdin chlorophyll protein (PERCP) and fluorescein isothiocyanate (FITC) antibodies: CD3, CD2,CD4/8, CD2/3/7, CD5/10/19, CD19/κ/λ, FMC7/CD23/CD19, CD38/56/19. All antibodies were purchased from Becton Dickinson (San José, CA) except for bcl-2, which was purchased from Pharmingen. After incubation red blood cells were lysed with ammonium chloride lysing solution (diluted to 10%) for 5 minutes and then washed twice. If small fragments were still present, the suspension was filtered through 50-micron filters; finally an equal part of 1% paraformaldehyde was added to each tube for cell fixation. When the routine technique failed to detect intra-cytoplasmatic light chains on the surface, cells were suspended in permeabilizing solution and incubated for 30’ in the dark. FC was then performed using a three-color analysis technique on a Becton Dickinson (San José, CA) FACS scan as previously described [9]. As far as the data evaluation is concerned, an antibody was considered expressed when a minimum of 10% of the gated cells were positive. Clonality assessment for T-cell process was established according to the ISCL/EORTC immunophenotypic criteria for the diagnosis of peripheral blood involvement by cutaneous T-cell lymphoma, because there are no similar criteria for FC in lymph node samples. The ISCL/EORTC criteria are: 1) >40% of CD + T-cells exhibit loss of CD7 or >30% of CD + T-cells exhibit loss of CD26, 2) CD4:CD8 ratio greater of 10:1, and/or 3) aberrant expression of multiple pant-cell surface markers [5,11,12]. In light chain evaluation, κ:λ ratios greater than 4:1 or 1:2 were considered as definite evidence of monoclonality [18-20]. In cases of equivocal results or technical difficulties, residual material, stored in a tube at 4°C, was suitable for further analysis within 24 hours from sampling.

### Immunocytochemistry

Immunocytochemical stains were performed in three cases, using additional 95° alcohol-fixed smears; the primary antibodies used were UCHL1 (CD45RO), CD3 and CD20 (1:100; Dakopatts, Glostrup, Denmark). This procedure has been previously described [8,9].

### Molecular analysis

#### DNA extraction

In the four cases processed, sufficient high molecular weight DNA was extracted from the cells using a commercially available kit (QIAamp DNA Mini Kit, QIAGEN) according to the manufacturer’s instructions. Samples were centrifuged at 1300 rpm for 20 minutes to expel the RNAlater TM. After discharge of the supernatant, 20 μl of Proteinase K were added to each sample and incubated at 50°C for 3 hours. After a second incubation at 70°C for 10’ in 200 μl of Buffer AL, 200 μl ethanol (96-100%) were added; the mixture was then loaded onto a QIAamp Spin Column and centrifuged at 8000 rpm for 1’. The column was then washed with 500 μl l Buffer AW1 by centrifugation at 8000 rpm for 1’ and with 500 μl l Buffer AW2 by centrifugation at 14000 rpm for 3’. When all the residual ethanol had been removed, DNA was eluted with 70 ml Buffer AE by centrifugation at 8000 rpm for 1 minute.

#### TCRγ molecular analysis

One hundred ng of genomic DNA from each patient were amplified using two multiplex mixes that independently target preserved framework regions of the variable and joining regions of TCR **γ** chain locus that flanks the antigen-binding, complementarity determining region 3 (CDR3). The limit of confidence in the detection of this assay is approximately one clone T-cell among one hundred normal cells. Mix 1 contained the primers Vγ1-8, while mix 2 contained the primers V**γ** 5-10-11-12. PCR was performed using GoTaq® Green Master Mix (Promega, cat n. M7123), as indicated by the manufacturer, supplied by 0.4 μM of each primer. PCR reactions were performed with a Veriti® Thermocycler (Applied Biosystems) by incubating samples at 94°C for 7’, followed by 45 cycles of 95°C for 1’, 55°C for 1’, 72°C for 1’. The final extension step was performed for 10’ at 72°C and the samples were then chilled to 4°C. The PCR products generated from TCRγ assay were identified using a standard gel electrophoresis with ethidium bromide staining. Clonality was indicated when one of the multiplex mixes generated clonal band(s) of almost 190 bp, with a normal distribution of product sizes between 159 and 260 nucleotides [23,24].

#### Heteroduplex analysis and Denaturing High Performance Liquid Chromatography (DHPLC) analysis

Heteroduplex analysis was performed on PCR products at high temperature and rapid re-annealing of the DNA strands by immediate temperature reduction. This procedure causes a large portion of DNA strands to bind incorrectly to other non-homologous strands, creating DNA loops that cause significant reduction in the DNA capacity to migrate through the DHPLC column. Therefore, in the clonal samples with a polyclonal background, the heteroduplex analysis caused an increase in their separation and allowed the identification of the clonal TCRs. In the following DHPLC analysis the clonality status of each product was evaluated by Denaturing High Performance Liquid Chromatography on a Transgenomic WaveTM System Model 3500HT (DHPLC, Transgenomic TM, Omaha, NE, USA) on a high resolution micropellicular matrix. Elution profiles were performed at 50°C (native DNA). Ten μl samples were injected into a preheated (50°C) C18 reversed-phase column with non-porous polystyrene-divinylbenzene particles (DNA Sep, Transgenomic). The injected sample was eluted with a linear acetonitrile gradient consisting of buffer A (0.1 mol/L TEAA) and buffer B (0.1 mol/L TEAA, 250 mL/L acetonitrile) with a 2% increase in buffer B per minute. PCR products were separated with a flow rate of 0.9 mL/min and retention time was measured online via ultraviolet absorbance at 254 nm in the elute. The resulting diagrams showed absorbance intensity in millivolts over the retention time in minutes (mV/min) after injection into the column. In this way, the DNA isolated from a heterogeneous population of polyclonal T-cells produces a Gaussian distribution (bell-shaped curve) of amplified product on HA or DHPLC analysis, whereas a monoclonal population will generate a single peak of elution.

#### Sequencing analysis

The PCR amplicons were sequenced in both directions by the Sanger method. The identification of rearranged genes was performed by comparing the sequences obtained with the germline sequences available in the IMGT® databases (http://imgt.cines.fr; European Bioinformatics Institute, Montpellier, France).

## Competing interests

The authors declare that they have no competing interests.

## Authors’ contributions

PZ, EV: conception and design, interpretation of data, given final approval of the version to be published; IC, ALP, LVSF, AV, GB, MP, FP, CS, acquisition of data, drafting the manuscript, PZ: critical revision. All authors read and approved to be published.

## Pre-publication history

The pre-publication history for this paper can be accessed here:

http://www.biomedcentral.com/1471-2407/14/8/prepub
